# Supplementary material for: Aging affects reprogramming of pulmonary capillary endothelial cells after lung injury in male mice
Source: Nat Commun. 2025 Aug 6;16:7234. doi: 10.1038/s41467-025-62431-4 (PMC12328796; doi:10.1038/s41467-025-62431-4)
Supplement: Supplementary file 1 — Supplementary Figures S1-S7 [file 41467_2025_62431_MOESM1_ESM.docx]

**
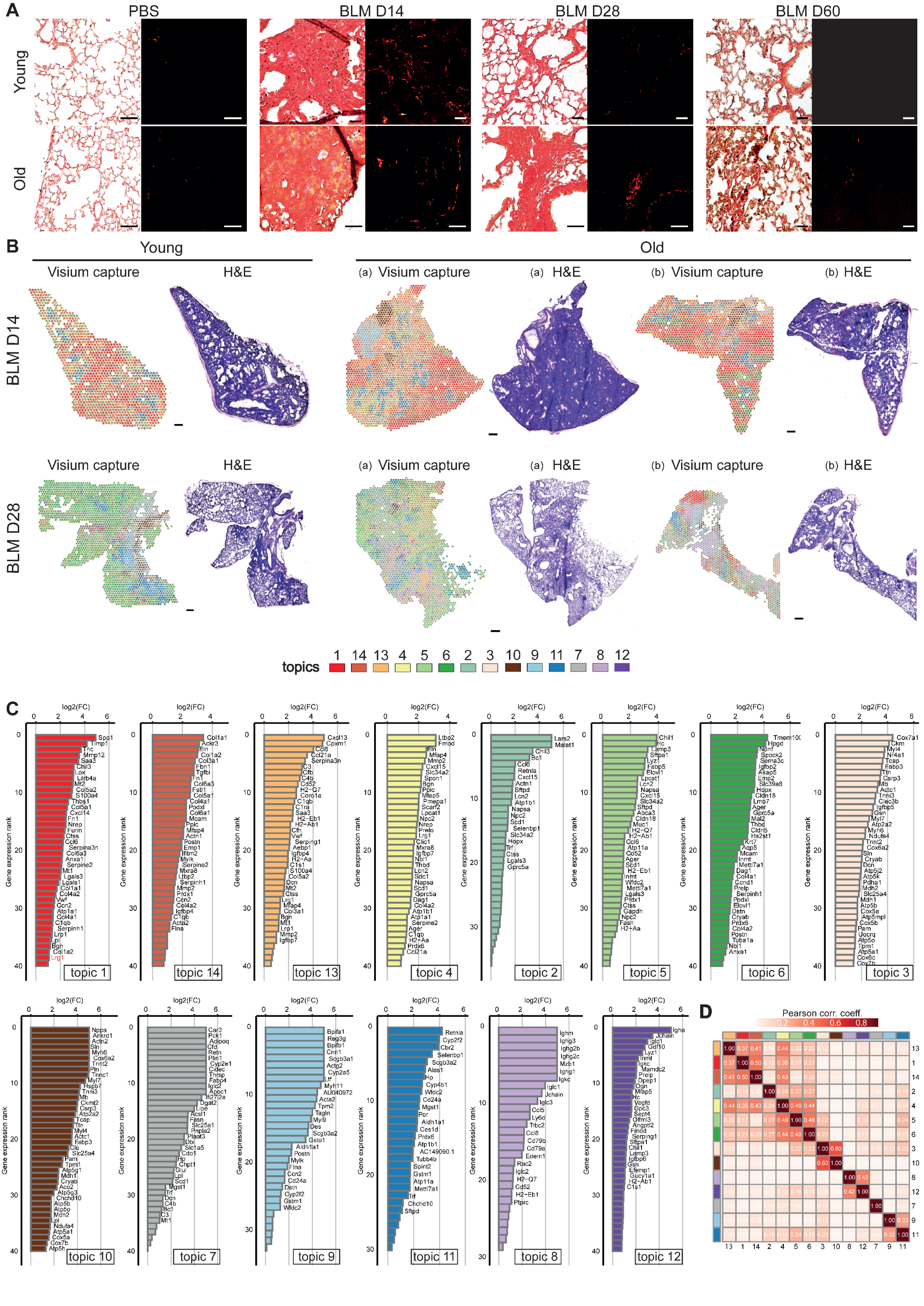
Supplemental Figure S1. Histological and spatial transcriptomics data integration of injured lung slices from young and old mice. (A)**Representative image of picrosirius red staining of collagen deposition on lung parenchyma from PBS or bleomycin (BLM)-treated young or old mice, at 14, 28 or 60 days after treatment under phase-contrast (left) or polarized light (right) microscopy. Scale bars=100µm.**(B)**Spatial transcriptomics data from histological sections (H&E) of lungs from young (n=1) and old (n=2) mice challenged with bleomycin and collected at fibrotic peak (day 14) and during regeneration (day 28). Spots are colored on the Visium capture according to assigned topics. Scale bars=200µm.**(C)**Top genes markers of each topics.**(D)** Heatmap of topics correlation based on gene expression.


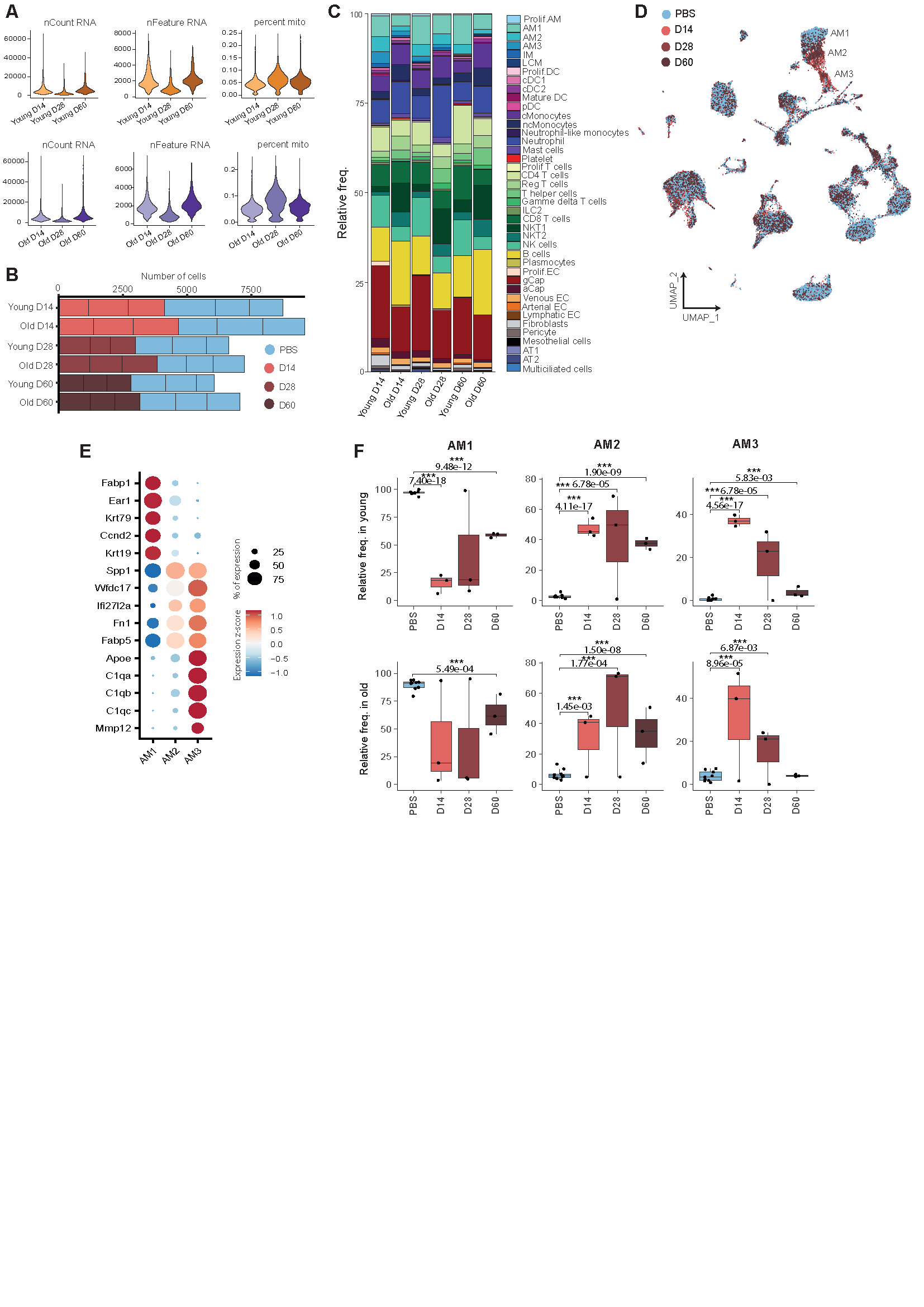


**Supplemental Figure S2. scRNA-seq analysis of young and aged mouse lungs following BLM challenge at 3 time points. (A)** Number of UMI, detected genes and percentage of mitochondrial content for each sequenced time point in young (top) and old (bottom) mice. **(B)** Number of cells by mice grouped by conditions, timepoints and age. **(C)** Relative proportions of all populations defined in integrated dataset. **(D)** UMAP of the integrated dataset. Cells are colored according to the timepoint. AM= alveolar macrophages. **(E)** Top genes markers in AM subpopulations (AM1, AM2 and AM3). **(F**) Relative proportions of alveolar macrophages subpopulations between young and old mice across time points. Source data are provided as a Source Data file. Statistic: P-values were calculated by Wald test and the Benjamini-Hochberg method for multiple tests correction.

**
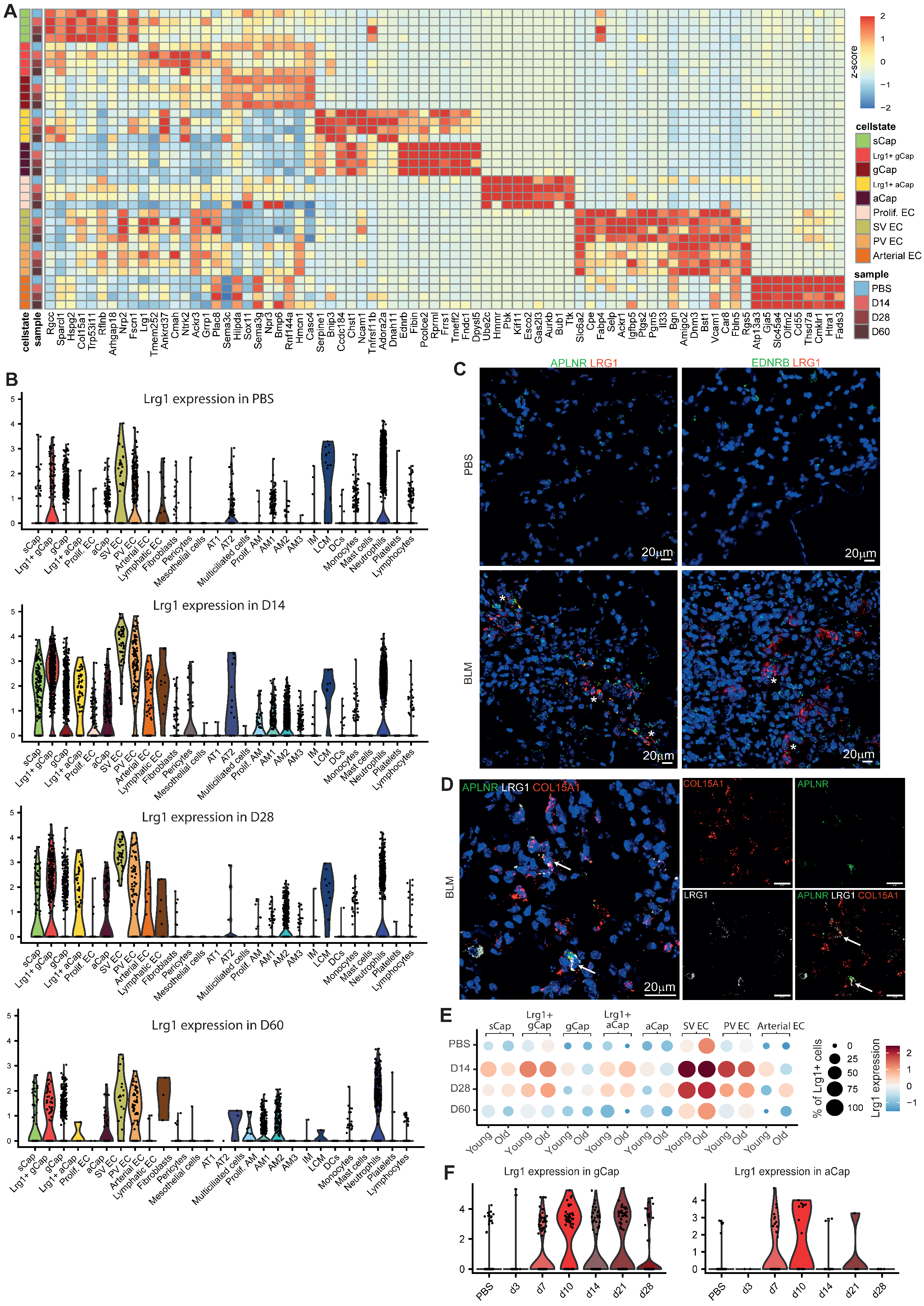
Supplemental Figure S3.** **Lrg1 expression in lung cell populations. (A)** Heatmap of top EC subpopulations markers. All cells from young and old mice from the same condition are pooled. Expression is indicated as a z-score. (**B)** Normalized expression of Lrg1 in all cell populations in PBS-treated mice and in bleomycin-treated mice at indicated time points. (**C-D)** *In situ* hybridization of Lrg1 mRNA with gCap marker *Aplnr* mRNA or aCap marker *Ednrb* mRNA **(C)** or sCap Markers *Col15a1* and *Aplnr* **(D)** in PBS- or BLM-treated lungs of young mice at D14. Representative image (n=3). Nuclei are counterstained with DAPI. **(E)** Normalized expression of Lrg1 comparison between pulmonary endothelial subpopulations and between young and old animals at indicated time points. **(F)** Normalized expression of Lrg1 in gCap and aCap isolated from Strunz et al. dataset (37).

**
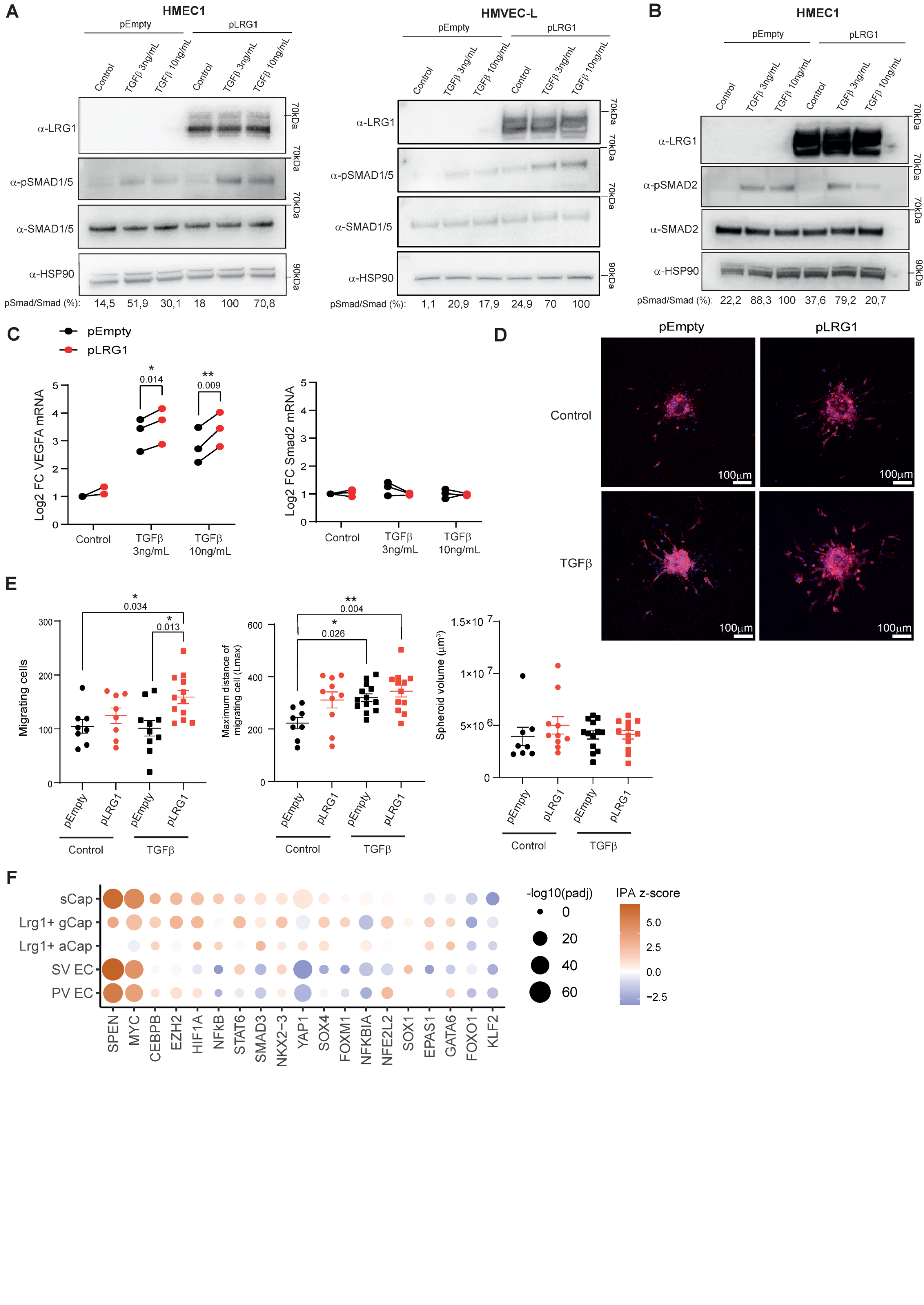
Supplemental Figure S4. LRG1 affects the TGF-β signaling towards a pro-angiogenic reponse.** HMEC1 and HMVEC-L were transduced with an empty or LRG1 (pLRG1) lentiviral particles. Transduced cells were stimulated 1 hour with TGF-β1 at 3 or 10 ng/mL or not (Control). **(A-B)** Western blot of pSmad1/5 and total Smad1/5 (**A**) or pSmad2 and total Smad2 (**B**) on protein lysates from pEmpty or pLRG1 cells under TGF-β1 stimulation. LRG1 and HSP90 protein level were revealed to validate the transduction efficacy and the loading, respectively. Experiments were conducted at least 3 times for HMEC1 and 2 times for HMVEC-L. Quantification of Phosphoprotein/total protein ratio for Smad1/5 or Smad2 in different experiments is indicated below the blot. Statistical analysis: ANOVA Two way with pairwise comparison (Bonferroni correction). (**C**) RT-qPCR of VEGFA and SMAD2 mRNA from pEmpty or pLRG1 HMEC-1 cells under TGF-β1 stimulation. Log2 fold change were normalized by RPLP0 expression and on pEmty control conditions. Experiments were conducted at least 3 times. Statistical analyses: Multiple paired t-test. **(D-E)** Endothelial spheroids made from pEmpty or pLRG1 HMVEC-L were embedded in collagen type I matrix and stimulated with TGF-β1 (10 ng/mL) for 18 h. Spheroids (n≥8) were labelled with phalloidin-594 and the nuclei were counterstained by DAPI. Experiments were conducted at least 3 times. Data are means ± SEM (**D**). The number of migrating cells and the maximum distance of migrating cells were quantified by image analysis software (FIJI) (**E**). Statistical analysis: ANOVA one way with pairwise comparison (Tukey correction). **(F)** Selection of top predicted upstream regulators based on the comparative Ingenuity pathway enrichment analysis of the different pathological signatures of Lrg1^pos^ PCEC subpopulations. Source data are provided as a Source Data file.

**
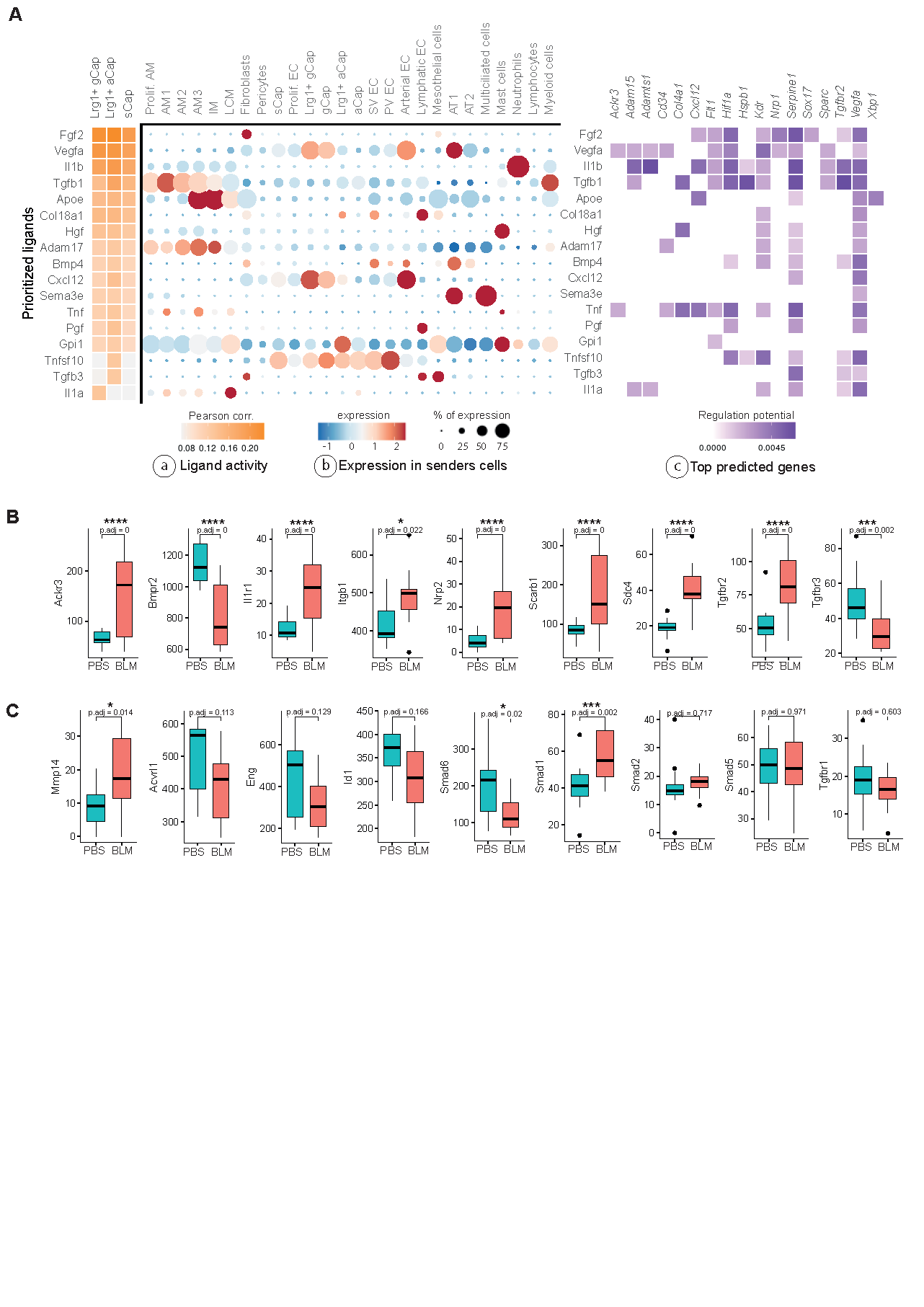
**

**Supplemental Figure S5. Bleomycin-induced PCEC subpopulations are associated with pro-angiogenic signalling. (A)** NicheNet analysis inferring upstream ligands most likely to induce a pro-angiogenic signature in Lrg1^pos^ EC subpopulations: (a) Prioritized ligands are ranked according to their potential activity; (b) Level and percentage of expression of the prioritized ligands; (c) Top predicted target genes of prioritized ligands. **(B)** Differentially-expressed receptors between bleomycin and PBS treated gCap. **(C)** Expression comparison between bleomycin and PBS treated gCap for *Mmp14*, Bmp9-associated signaling genes *Acvrl1, Eng, Id1* and *Smad6*, as well as *Smad1*, *Smad2*, *Smad5* and *Tgfbr1*. Source data are provided as a Source Data file. Statistic: P-values were calculated by Wald test and the Benjamini-Hochberg method for multiple tests correction.


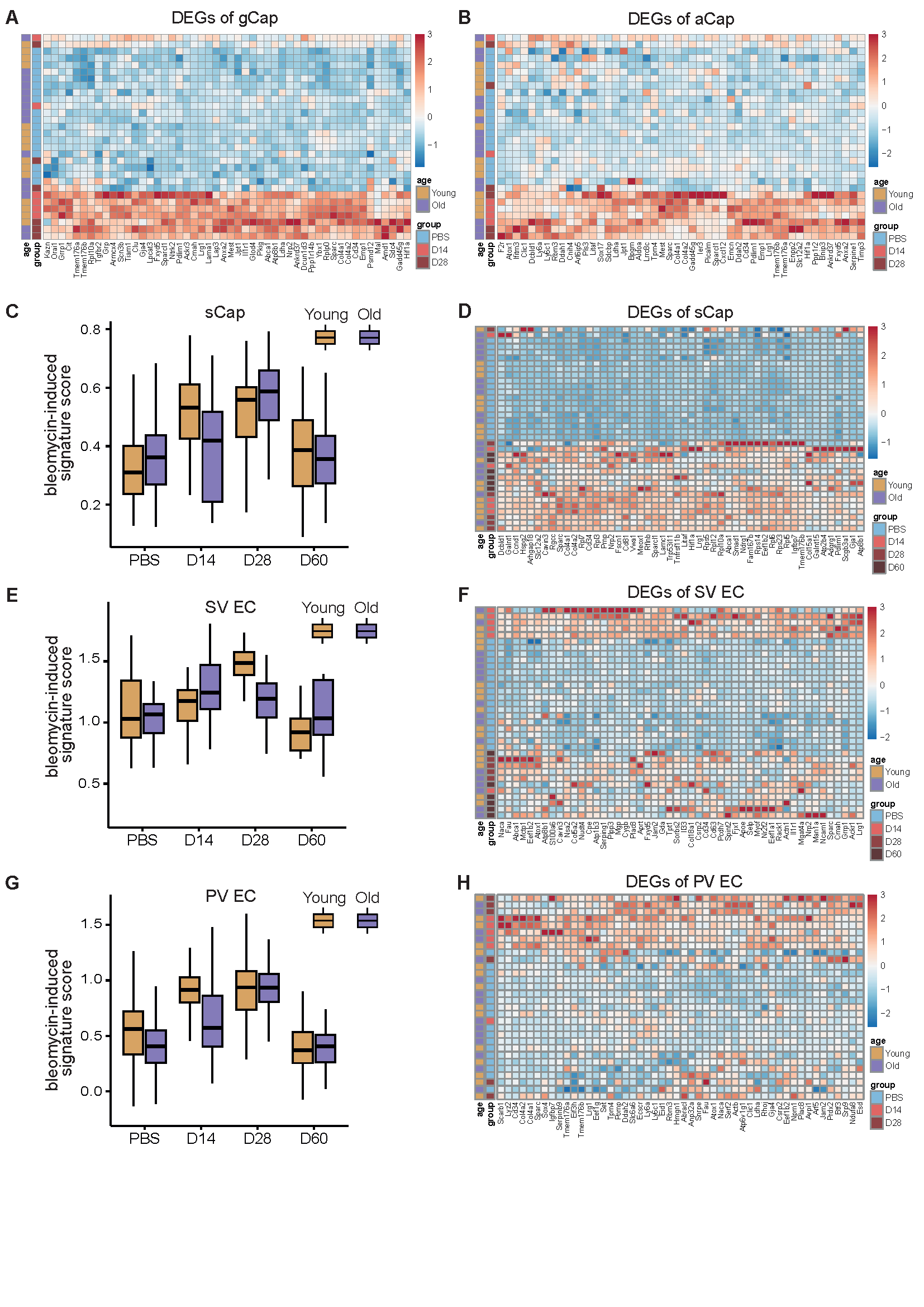


**Supplemental Figure S6. Expression of bleomycin-associated signatures in PCEC and venous EC from young and aged mouse lungs. (A-B)**Heatmap of top 50 DEGs between BLM and PBS treated-mice for gCap **(A)**or aCap **(B)**. **(C-H)** BLM-induced signature score in young and old mice across time points (**C, E, G**, see material and methods section) and heatmap of top 50 DEGs between BLM and PBS treated mice (**D, F, H**) for sCap (**C-D**), SV EC (**E-F**) or PV EC (**G-H**). Source data are provided as a Source Data file.

**
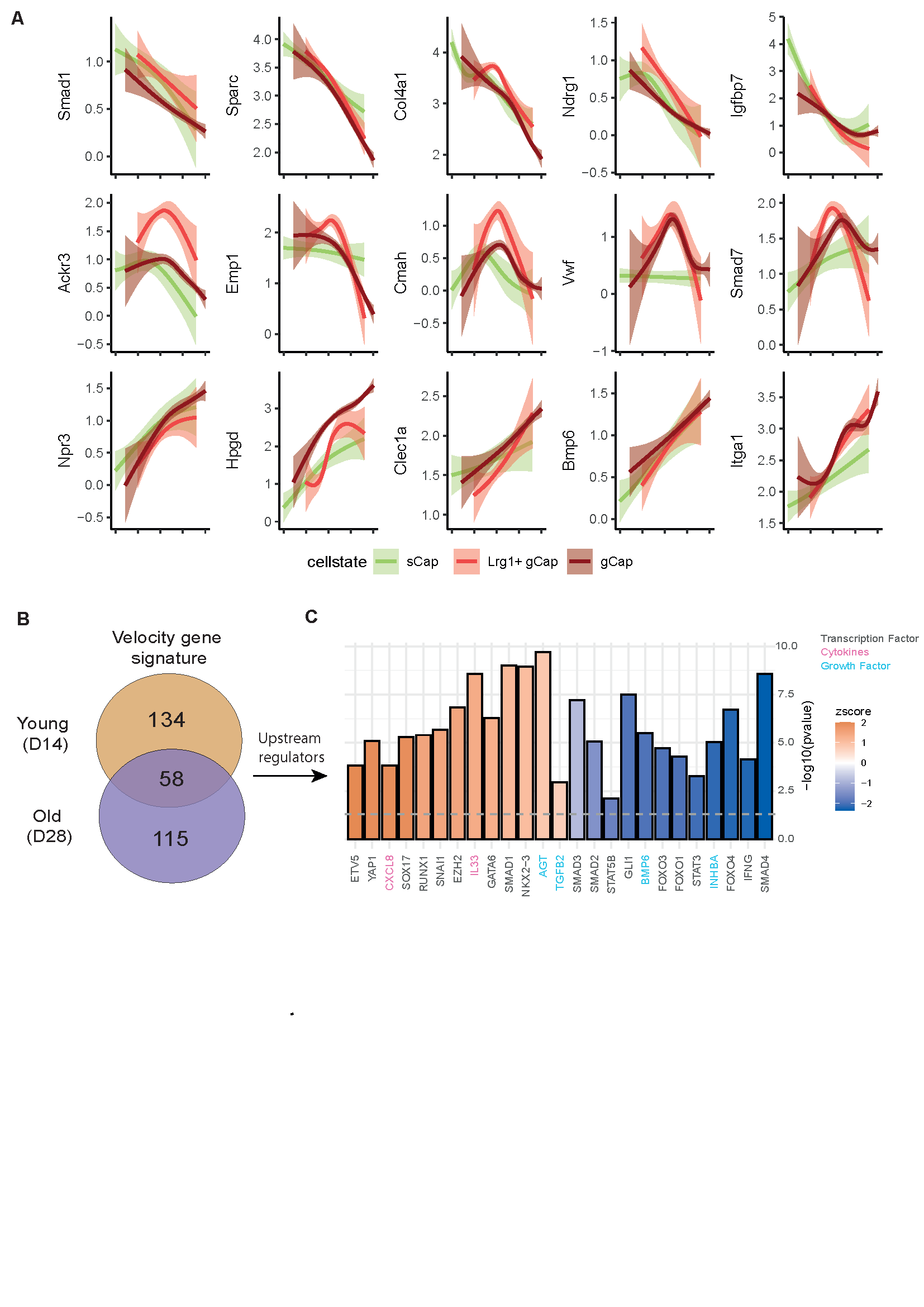
Supplemental Figure S7. Expression and functional analysis of selected genes according to RNA Velocity latent time analysis of PCEC subpopulations (sCap, Lrg1^pos^ gCap and gCap*).*** **(A)** Graphs showing expression pattern of selected genes according to Velocity latent time. The cell population (cellstate: sCap, Lrg1^pos^ gCap and gCap) are indicated with different colors. **(B)** Venn diagram showing the distinct and common genes used in the RNA Velocity analysis to model trajectory between PCEC populations for young (day 14) and old (d28) mice. **(C)** Selection of top predicted upstream regulators enriched in the common signature of 58 genes (see Supplemental table S7) between young and old mice.
