# Supplementary material for: Aging affects reprogramming of pulmonary capillary endothelial cells after lung injury in male mice
Source: Nat Commun. 2025 Aug 6;16:7234. doi: 10.1038/s41467-025-62431-4 (PMC12328796; doi:10.1038/s41467-025-62431-4)
Supplement: Supplementary file 9 — Reporting Summary [file 41467_2025_62431_MOESM9_ESM.pdf]

nature portfolio

Corresponding author(s): *Double-anonymous peer review submissions: write DAPR and your manuscript number here instead of author names.*

Last updated by author(s): *YYYY-MM-DD*



# Reporting Summary

Nature Portfolio wishes to improve the reproducibility of the work that we publish. This form provides structure for consistency and transparency in reporting. For further information on Nature Portfolio policies, see our Editorial Policies and the Editorial Policy Checklist.

## Statistics

For all statistical analyses, confirm that the following items are present in the figure legend, table legend, main text, or Methods section.

| n/a | Confirmed | |
|---|---|---|
| ☐ | ☒ | The exact sample size (*n*) for each experimental group/condition, given as a discrete number and unit of measurement |
| ☐ | ☒ | A statement on whether measurements were taken from distinct samples or whether the same sample was measured repeatedly |
| ☐ | ☒ | The statistical test(s) used AND whether they are one- or two-sided<br>*Only common tests should be described solely by name; describe more complex techniques in the Methods section.* |
| ☐ | ☒ | A description of all covariates tested |
| ☐ | ☒ | A description of any assumptions or corrections, such as tests of normality and adjustment for multiple comparisons |
| ☐ | ☒ | A full description of the statistical parameters including central tendency (e.g. means) or other basic estimates (e.g. regression coefficient) AND variation (e.g. standard deviation) or associated estimates of uncertainty (e.g. confidence intervals) |
| ☐ | ☒ | For null hypothesis testing, the test statistic (e.g. *F*, *t*, *r*) with confidence intervals, effect sizes, degrees of freedom and *P* value noted<br>*Give P values as exact values whenever suitable.* |
| ☒ | ☐ | For Bayesian analysis, information on the choice of priors and Markov chain Monte Carlo settings |
| ☒ | ☐ | For hierarchical and complex designs, identification of the appropriate level for tests and full reporting of outcomes |
| ☐ | ☒ | Estimates of effect sizes (e.g. Cohen's *d*, Pearson's *r*), indicating how they were calculated |

*Our web collection on statistics for biologists contains articles on many of the points above.*

## Software and code

Policy information about availability of computer code

| | |
|---|---|
| Data collection | Data were collected on the following instruments: Illumina NextSeq 2000, Microscope: LSM780 Zeiss, Axio Scan Z1 / Axioscan 7 scanner Zeiss, Real-Time PCR system: LightCycler 480 Roche, (GSE135893, GSE141259) |
| Data analysis | Software and tools used for data analysis: Graphpad Prism 8.4.3 (la Jolla, CA, USA), ImageJ, cellranger 3.0.2 (10X Genomics), CITE-seq-Count 1.4.2, kallisto 0.46.2, spaceranger 1.3.1 (10X Genomics), R 4.0.2, Python 3.8, Ingenuity Pathway analysis (IPA, Ingenuity® Systems, www.ingenuity.com) |

For manuscripts utilizing custom algorithms or software that are central to the research but not yet described in published literature, software must be made available to editors and reviewers. We strongly encourage code deposition in a community repository (e.g. GitHub). See the Nature Portfolio guidelines for submitting code & software for further information.

## Data

Policy information about availability of data

All manuscripts must include a data availability statement. This statement should provide the following information, where applicable:

- Accession codes, unique identifiers, or web links for publicly available datasets
- A description of any restrictions on data availability
- For clinical datasets or third party data, please ensure that the statement adheres to our policy

The scRNA-seq and spatial transcriptomic data sets have been deposited in the Gene Expression Omnibus SuperSerie GSE234199. Code availability. The scripts used

for the analysis of scRNA-seq and spatial transcriptomic data are available on github :
https://github.com/marintruchi/Aging_affects_reprogramming_of_PCEC

## Research involving human participants, their data, or biological material

Policy information about studies with human participants or human data. See also policy information about sex, gender (identity/presentation), and sexual orientation and race, ethnicity and racism.

| | |
|---|---|
| Reporting on sex and gender | Sex and/or gender was not considered in the study design. Both sexes were included in the study and distributed randomly in the experiments. We did not observe any sex-dependent differences in the data. Due to the limited number of patient samples used in the analysis, statistical adjustment for sex or gender analysis was not performed. In this study, we used lung tissues from five normal donors (two females and three males) and five patients with IPF (two females and three males). |
| Reporting on race, ethnicity, or other socially relevant groupings | Race and ethnicity were not taken into account in the study. |
| Population characteristics | N/A |
| Recruitment | N/A |
| Ethics oversight | Lung tissue sections were obtained from the UGMLC Giessen Biobank affiliated to the European IPF Registry as well as from the Nice Hospital-Integrated Biobank (transfer authorization from the French ministry of research n° AC-2021-4655). |

Note that full information on the approval of the study protocol must also be provided in the manuscript.

# Field-specific reporting

Please select the one below that is the best fit for your research. If you are not sure, read the appropriate sections before making your selection.

☒ Life sciences ☐ Behavioural & social sciences ☐ Ecological, evolutionary & environmental sciences

For a reference copy of the document with all sections, see nature.com/documents/nr-reporting-summary-flat.pdf

# Life sciences study design

All studies must disclose on these points even when the disclosure is negative.

| | |
|---|---|
| Sample size | Sample sizes were determined based on prior studies performed in the field and the number of replicates for statistical analyses are specified in the figure legends. |
| Data exclusions | No data were excluded from the experiments presented in this study, with the exception of 2 samples in the spatial transcriptomics experiment (Visium), which appeared unusable due to a very low level of genes detected per spot , as described in the Method section. |
| Replication | Figure legends indicate the number of mice (in vivo experiments) and the number of biologically independent replicates (in vitro experiments). All animal studies were independently performed twice. In vitro experiments were performed three times. |
| Randomization | For each experiment, animais were of identical genotypes, sex, weight and age and were randomly assigned to experimental groups. |
| Blinding | In animal experiments, investigators were not blinded to group allocation. Blinding was not used during scRNA-seq experiment but samples from control and bleomycin-treated mice were multiplexed and a single library was been made for each time point. There was no blinding in cell culture experiments. |

# Reporting for specific materials, systems and methods

We require information from authors about some types of materials, experimental systems and methods used in many studies. Here, indicate whether each material, system or method listed is relevant to your study. If you are not sure if a list item applies to your research, read the appropriate section before selecting a response.

## Materials & experimental systems

| n/a | Involved in the study |
| --- | --- |
| ☐ | ☒ Antibodies |
| ☐ | ☒ Eukaryotic cell lines |
| ☒ | ☐ Palaeontology and archaeology |
| ☐ | ☒ Animals and other organisms |
| ☒ | ☐ Clinical data |
| ☒ | ☐ Dual use research of concern |
| ☒ | ☐ Plants |

## Methods

| n/a | Involved in the study |
| --- | --- |
| ☒ | ☐ ChIP-seq |
| ☒ | ☐ Flow cytometry |
| ☒ | ☐ MRI-based neuroimaging |

# Antibodies

| | |
| --- | --- |
| Antibodies used | All antibodies used in this study are commercially available and described in the methods.The following antibodies were used in immunofluorescence experiments: Mouse Anti-Human CD31 (MA5-13188 Thermofisher, 1:50), Rabbit Anti-Human COL15A1 (53667 Invitrogen, 1:100), Purified Mouse Anti- αSMA–FITC (F3777 Sigma-Aldrich, 1:500), donkey DyLight 594 Anti-Rabbit IgG (A32754 Life Technologies, 1:500), donkey DyLight 647 Anti-Mouse IgG (A31571 Life Technologies, 1:500). The following antibodies were used in western blot experiments: Rabbit anti-Human pSmad2 (18338, Cell signaling, 1:1000), Rabbit anti-Human Smad2 (5339, Cell signaling, 1:1000), Rabbit anti-Human pSmad1/5 (9516, Cell signaling, 1:1000),Rabbit anti-Human Smad5 (9517, Cell signaling, 1:1000), Rabbit anti-Human LRG1 (PA5-96832, Thermofisher, 1:1000), Rabbit anti-Human HSP90 (4877, Cell signaling, 1:3000) and Goat anti-rabbit HRP (AP0448, Dako, 1:10000). |
| Validation | All antibodies were validated by the manufacturer and have multiple citations for the relevant cell type. Investigators independently validated the selected antibodies by reproducing published findings. |

# Eukaryotic cell lines

Policy information about cell lines and Sex and Gender in Research

| | |
| --- | --- |
| Cell line source(s) | Human lungs primary microvascular endothelial cells (HMVEC-L, male, batch ID:23TL178219) were obtained from Lonza (CC-2527, Basel, Switzerland) and HMEC1 were obtained by a collaborator (Christian Dani Team, Université Côte d'Azur, CNRS, INSERM, iBV, Nice, France). |
| Authentication | Cells were not authenticated |
| Mycoplasma contamination | Cells were tested and were mycoplasma free. |
| Commonly misidentified lines (See ICLAC register) | No commonly misidentified lines were used |

# Animals and other research organisms

Policy information about studies involving animals; ARRIVE guidelines recommended for reporting animal research, and Sex and Gender in Research

| | |
| --- | --- |
| Laboratory animals | Seven-week and 18-month-old C57BL/6 male mice were purchased from Charles River. |
| Wild animals | No wild animais were used |
| Reporting on sex | All experiments were conducted on male mice. This decision was based on the specific aim of our study (aging) and on the observation that human fibrosis is an age-associated disease, with approximately 70% of IPF cases occurring in men. Consequently, the use of male animals in our study was intended to better reflect the nature of age-associated disease and its prevalence in men. |
| Field-collected samples | N/A |
| Ethics oversight | All animal care and experimental protocols were conducted according to European, national and institutional regulations (Protocol numbers: 00236.03 and APAFIS#31298, IPMC approval E0615252; Protocol Number APAFIS#12540, University of Lille; approval 00236.03 CNRS). Personnel from the laboratory performed all experimental protocols under strict guidelines to ensure careful and consistent handling of the mice. |

Note that full information on the approval of the study protocol must also be provided in the manuscript.

## Plants

| | |
|---|---|
| Seed stocks | N/A |
| Novel plant genotypes | N/A |
| Authentication | N/A |
