# Supplementary material for: Aging affects reprogramming of pulmonary capillary endothelial cells after lung injury in male mice
Source: Nat Commun. 2025 Aug 6;16:7234. doi: 10.1038/s41467-025-62431-4 (PMC12328796; doi:10.1038/s41467-025-62431-4)
Supplement: Supplementary file 11 — Supplementary Information [file 41467_2025_62431_MOESM11_ESM.docx]

**Supplementary information**

**Correspondence of supplementary tables:**

**Supplementary table S1.** Differential expression analysis between topics deconvoluted from spatial transcriptomic data.

**Supplementary table S2.** Differential expression analysis (Seurat) between annotated subpopulations of single cell RNA-seq data.

**Supplementary table S3.** Differential expression analysis (DESeq2) between lung cell types from bleomycin-treated and PBS-treated mice.

**Supplementary table S4.** Differential expression analysis (DESeq2) between IPF patients and healthy control for aCap, gCap, sCap, SV EC and PV EC, from the Habermann et al. Dataset^34^.

**Supplementary table S5.** Differential expression analysis (DESeq2) between old and young aCap and gCap cells in fibrotic condition.

**Supplementary table S6.** Differential expression analysis (DESeq2) between old and young aCap and gCap cells in physiological condition.

**Supplemental table S7.** List of common genes selected in the RNA Velocity analysis to model trajectory between PCEC populations for young (day 14) and old (d28) mice.

**Correspondence of supplemental Figures:**

**Supplemental Figure S1. Histological and spatial transcriptomics data integration of injured lung slices from young and old mice.**

**Supplemental Figure S2. scRNA-seq analysis of young and aged mouse lungs following BLM challenge at 3 time points.**

**Supplemental Figure S3.** **Lrg1 expression in lung cell populations.**

**Supplemental Figure S4. LRG1 affects the TGF-β signalling towards a pro-angiogenic response.**

**Supplemental Figure S5. Bleomycin-induced PCEC subpopulations are associated with pro-angiogenic signaling.**

**Supplemental Figure S6. Expression of bleomycin-associated signatures in PCEC and venous EC from young and aged mouse lungs.**

**Supplemental Figure S7. Expression and functional analysis of selected genes according to RNA Velocity latent time analysis of PCEC subpopulations**.
