# Supplementary figures and images for: Aging affects reprogramming of pulmonary capillary endothelial cells after lung injury in male mice
Source: Nat Commun. 2025 Aug 6;16:7234. doi: 10.1038/s41467-025-62431-4 (PMC12328796; doi:10.1038/s41467-025-62431-4)

**Figure S4A**  
**Left (HMEC1) /**

**Blot 1**

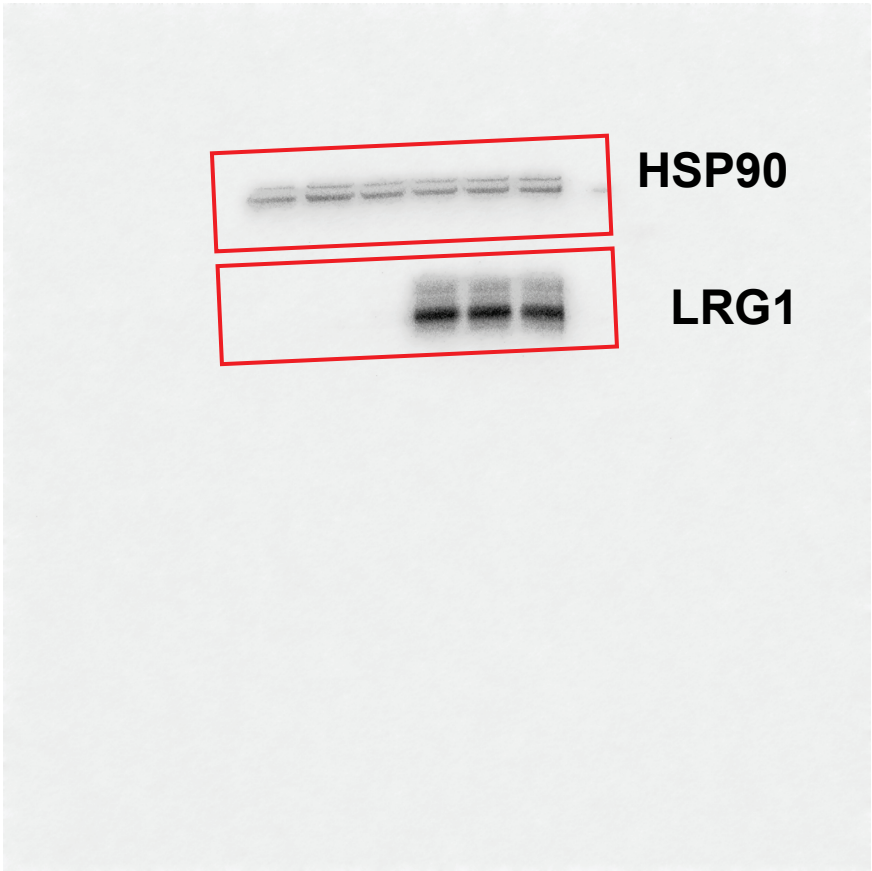

**Blot 2**

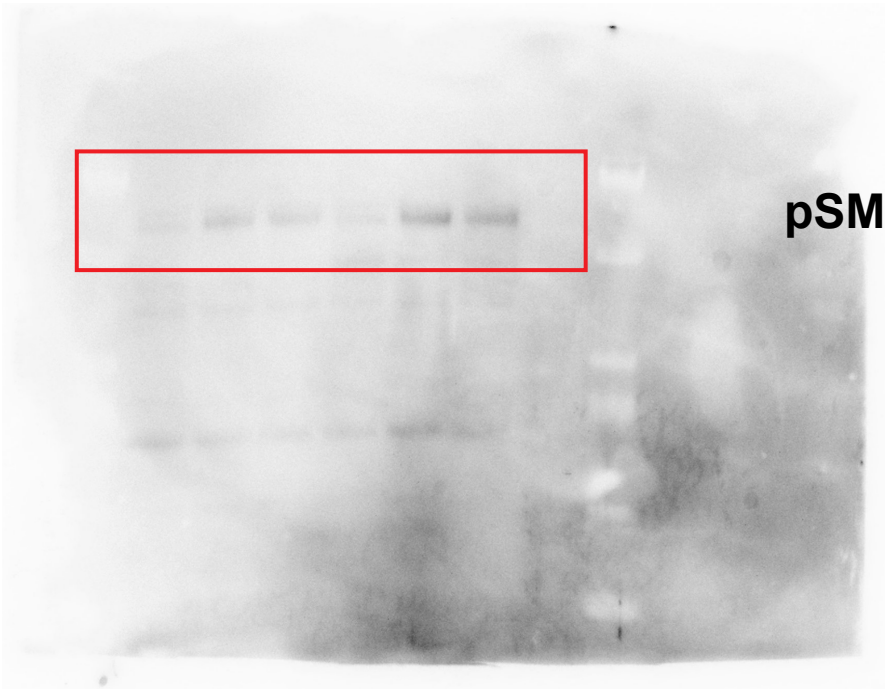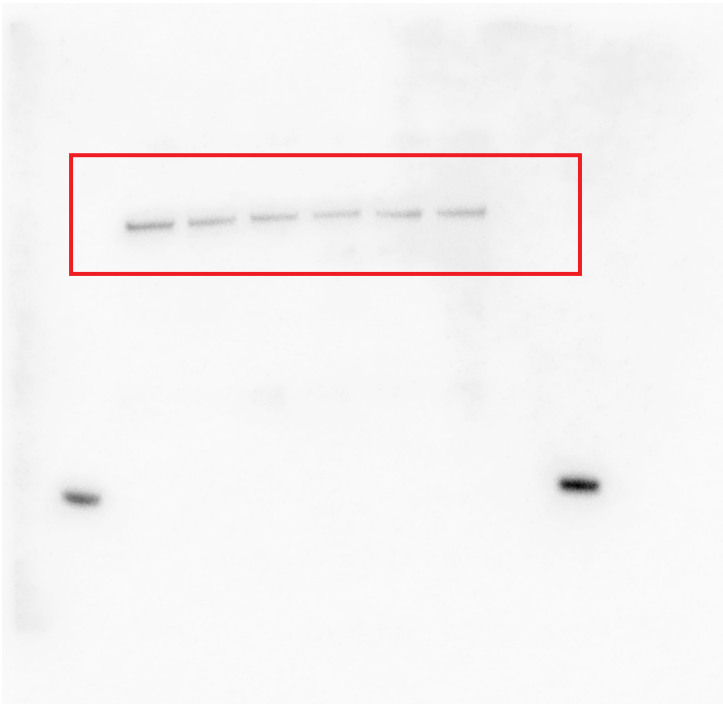

**Figure S4A**  
**Right (HMVEC-L) /**

**Blot 1**

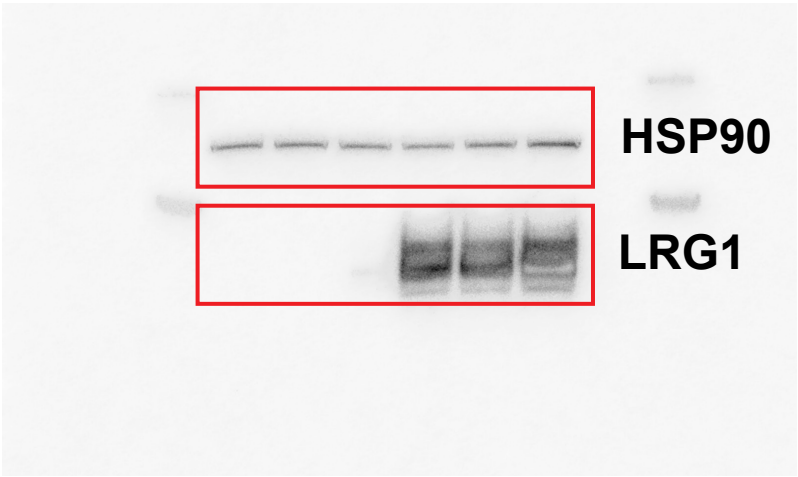

**Blot 2**

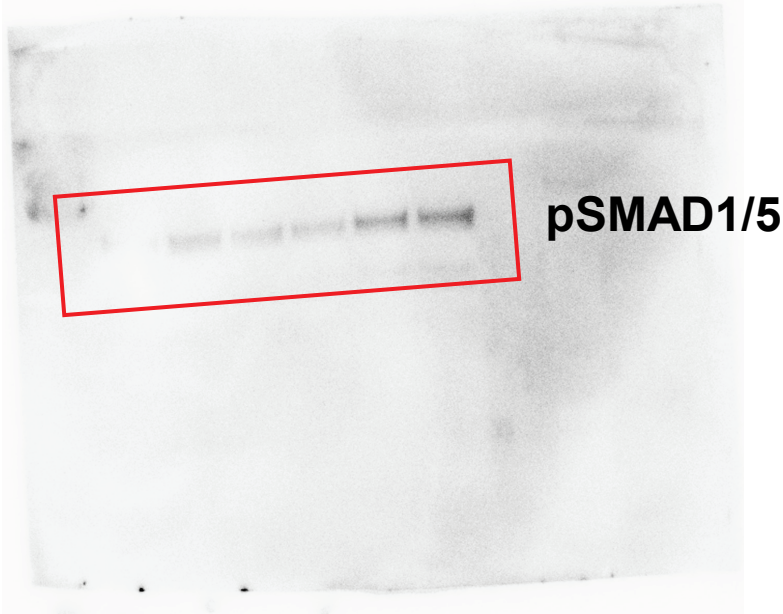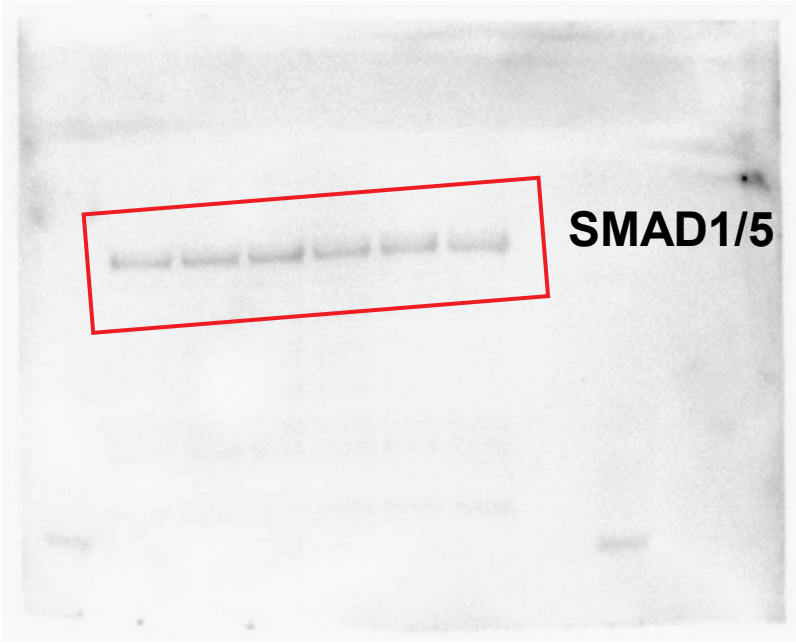

**Figure S4B**

**Blot 1**

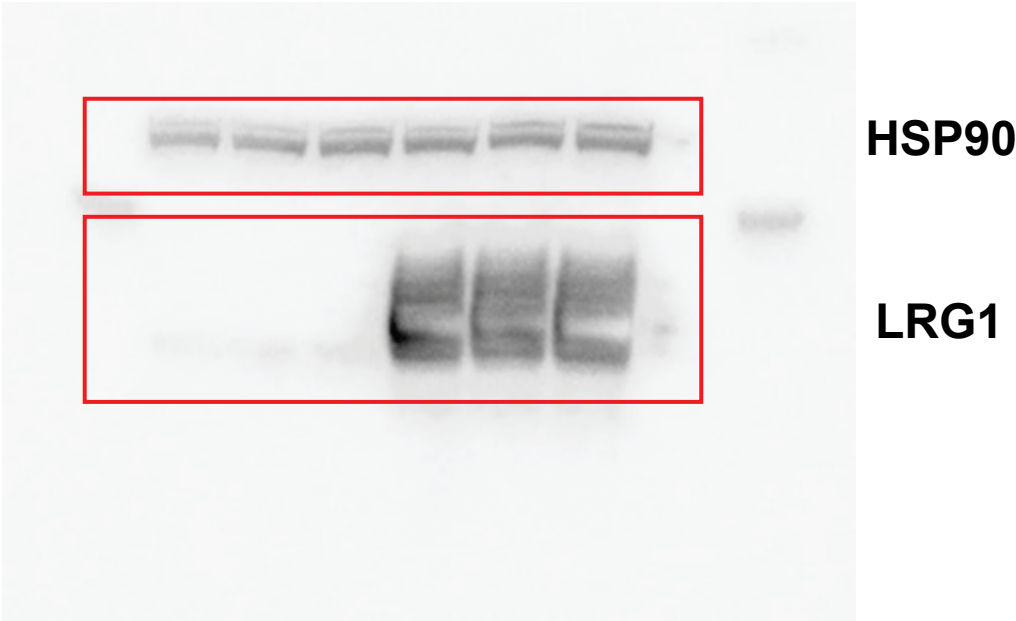

**Blot 2**

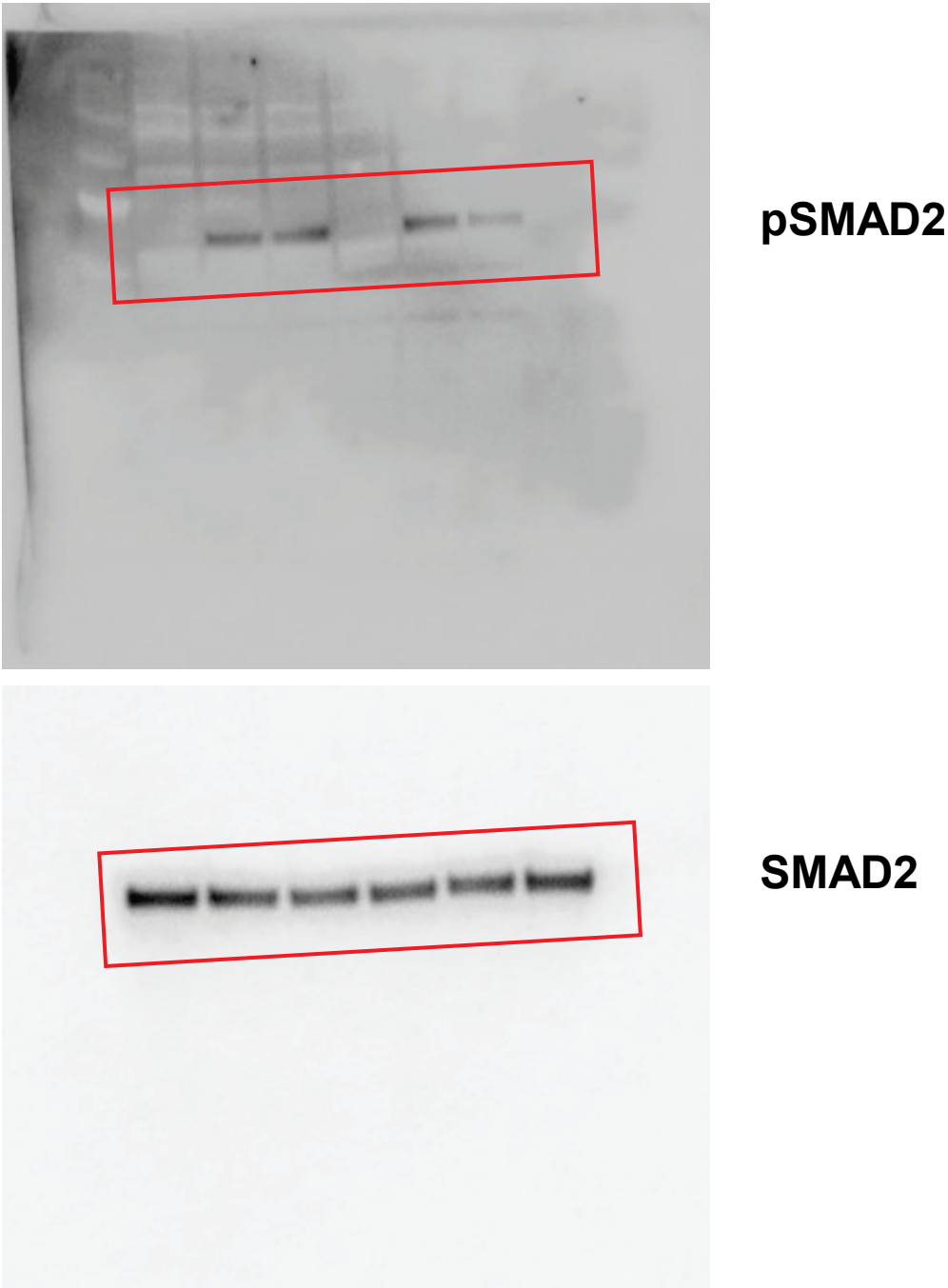

Supplement: Supplementary file 12 — Source Data 1 [file 41467_2025_62431_MOESM12_ESM.pdf]
